# Lipid Motif of a Bacterial Antigen Mediates Immune Responses via TLR2 Signaling

**DOI:** 10.1371/journal.pone.0019781

**Published:** 2011-05-17

**Authors:** Amit A. Lugade, Anna Bianchi-Smiraglia, Vandana Pradhan, Galina Elkin, Timothy F. Murphy, Yasmin Thanavala

**Affiliations:** 1 Department of Immunology, Roswell Park Cancer Institute, Buffalo, New York, United States of America; 2 Department of Cancer Genetics, Roswell Park Cancer Institute, Buffalo, New York, United States of America; 3 Department of Medicine, University at Buffalo, State University of New York, Buffalo, New York, United States of America; Wayne State University, United States of America

## Abstract

The cross-talk between the innate and the adaptive immune system is facilitated by the initial interaction of antigen with dendritic cells. As DCs express a large array of TLRs, evidence has accumulated that engagement of these molecules contributes to the activation of adaptive immunity. We have evaluated the immunostimulatory role of the highly-conserved outer membrane lipoprotein P6 from non-typeable *Haemophilus influenzae* (NTHI) to determine whether the presence of the lipid motif plays a critical role on its immunogenicity. We undertook a systematic analysis of the role that the lipid motif plays in the activation of DCs and the subsequent stimulation of antigen-specific T and B cells. To facilitate our studies, recombinant P6 protein that lacked the lipid motif was generated. Mice immunized with non-lipidated rP6 were unable to elicit high titers of anti-P6 Ig. Expression of the lipid motif on P6 was also required for proliferation and cytokine secretion by antigen-specific T cells. Upregulation of T cell costimulatory molecules was abrogated in DCs exposed to non-lipidated rP6 and in TLR2^−/−^ DCs exposed to native P6, thereby resulting in diminished adaptive immune responses. Absence of either the lipid motif on the antigen or TLR2 expression resulted in diminished cytokine production from stimulated DCs. Collectively; our data suggest that the lipid motif of the lipoprotein antigen is essential for triggering TLR2 signaling and effective stimulation of APCs. Our studies establish the pivotal role of a bacterial lipid motif on activating both innate and adaptive immune responses to an otherwise poorly immunogenic protein antigen.

## Introduction

The initiation of a robust and long-lasting immune response to infections and vaccination is thought to depend on effective TLR mediated recognition and signaling on innate immune cells. TLR stimulation in innate immune cells, such as dendritic cells and macrophages, activates various cytokine genes that instruct the nature of the ensuing T cell and B cell response [1]. The innate immune cell itself is influenced by the TLR signal and results in upregulation of T cell co-stimulatory molecules and secretion of proinflammatory cytokines. The nature of this response orchestrates the magnitude and quality of the ensuing T cell and B cell response, thus effective vaccination requires potent TLR activation [2]. Vaccines against bacterial pathogens utilize conserved outer membrane antigens, which may also serve as TLR ligands [3], [4]. The extent to which a given vaccine antigen induces potent and sustained immune responses is likely to be dependent on whether it or the adjuvant can stimulate both innate and adaptive immunity. This study has examined the TLR mediated augmentation of innate and adaptive immune responses to a candidate vaccine antigen for a respiratory pathogen.

Nontypeable *Haemophilus influenzae* (NTHI) is a commensal gram-negative coccobacillus that resides in the human upper respiratory tract and causes recurring episodes of infections in patients with chronic obstructive pulmonary disease (COPD) and children with otitis media. Current research efforts are evaluating the efficacy of several NTHI gene products as candidate vaccine antigens [5]. These include the major outer membrane proteins (P1, P2, P4, P5), adhesins, and lipoolgosaccharide. Each of the candidate antigens tested have elicited IgA and IgG following immunizations in murine, rat, and chinchilla models [6], [7]. Protection from NTHI colonization by increased clearance of the bacteria and reduction in accumulation of middle ear fluids reveal the functional capacity of vaccination against molecules expressed by NTHI. Antigenic heterogeneity in many of the surface molecules in NTHI strains suggests that a highly conserved, immunogenic molecule is required for formulation of an effective vaccine.

Outer membrane protein 6 (P6) is a 16 kDa lipoprotein highly conserved at the nucleotide and amino acid level among all tested strains of NTHI [8]. This lipoprotein functions as an anchor between the outer membrane and the bacterial cell due to its association with peptidoglycan. In addition to high sequence homology between strains, P6 also expresses epitopes on the outer membrane accessible for antibody binding. In various models of NTHI infection antibody responses to P6 were associated with protection [9], [10]. We have previously demonstrated that T cell responses to P6 are associated with relative protection against NTHI infection in adults with COPD [11]. As a lipoprotein, P6 expresses a tripalmitoyl lipid motif at the N-terminus, a common motif among bacterial lipoprotein family members [12]. The presence of this lipid motif permits recognition of P6 by TLR2, whose expression is found on macrophages, dendritic cells, B cells, neutrophils, mast cells and endothelial cells [12]. The induction of TLR2 signalling by its ligands leads to the production of proinflammatory cytokines and mucin via NF-κB activation. The immunogenic nature of this highly conserved lipoprotein makes P6 a promising vaccine candidate for NTHI and prompted our evaluation of the lipid component's requirement and its contribution to the immunogenicity of P6.

There is compelling evidence supporting the role of some lipid motifs to act as adjuvants and potentiate immune responses to otherwise poorly immunogenic proteins. Synthetic lipid motifs coupled to peptides representing influenza viral epitopes recognized by CD8^+^ and CD4^+^ T cells elicit protective immune responses. Bacterially derived lipid motifs were the strongest immunogens. In addition, the immunogenicity of these synthetic lipopeptides was dependent on the position of the lipid insert onto the peptide. In order to empirically test the importance of the lipid motif on the generation of anti-P6 responses, a recombinant P6 variant lacking the tripalmitoyl lipid motif was generated. Therefore, one important goal of this study was to evaluate the immunogenic potential of a non-lipidated form of P6, which still retains expression of a T cell helper epitope. Our studies provide a detailed analysis of the pivotal role of the lipid motif in the immunogenicity of P6 and elicitation of anti-P6 antibodies and T cells *in vivo*.

## Material and Methods

### Mice

Female C57BL/6NCr (WT) mice were purchased from NCI. Female B6.129-*Tlr2^tm1Kir^*/J (H2^b^) (TLR2^−/−^) mice were purchased from Jackson Laboratories (Bar Harbor, ME). Mice were maintained under specific pathogen free conditions at Roswell Park Cancer Institute and all procedures performed on animals were approved by the Institutional Animal Care and Use Committee, and complied with all state, federal, and NIH regulations.

### Native P6 Purification

Purification of the protein P6 from NTHI strain 1479 was performed as previously described [9], [13]. The extracted native P6 migrated as a single 16-kDa band in SDS-PAGE and was assayed with PYROGENT-5000 (Lonza) to ensure no endotoxin contamination was present in the purified protein.

### Recombinant non-lipidated P6 purification

To generate purified recombinant P6 that lacks the N-terminal tripalmitoyl lipid motif, the P6 gene was cloned into plasmid pDEST17 (Invitrogen), which expresses the P6 protein with an N-terminal 6× histidine tag. Recombinant non-lipidated P6 was purified by affinity chromatography and elution from a Talon column (BD Biosciences) as previously described [14]. The purified protein migrated as a single band in SDS-PAGE and was further assayed to ensure no endotoxin contamination was present.

### BMDC purification

Bone marrow from femurs and tibias of WT or TLR2^−/−^ mice was flushed out with a 21 gauge needle and complete medium (RPMI-1640 supplemented with 4 mM glutamine, 150 µg/ml penicillin-streptomycin, 0.2 mM non-essential amino acids, 20 mM HEPES, 1 mM sodium pyruvate, 50 µg/ml gentamycin, 50 µM 2-β-mercaptoethanol, 10% FCS). The bone marrow cells were cultured in 6 well plates at a concentration of 2×10^6^ cells/well in presence of 20 ng/ml of GM-CSF and 10 ng/ml of murine rIL-4 (BD Pharmingen). Media was changed every 2 days and on day 6 of culture, DCs were activated overnight with 300 ng/ml of either purified native P6, non-lipidated rP6, or 2×10^7^ CFU/ml formalin-killed NTHI. Media alone controls were also included. Cells were analyzed next day by flow cytometry for cell surface markers or used in a P6-specific T cell assays.

### Flow cytometry

Cells were washed in FACS staining buffer (0.9 g/L NaN_3_, 5% FBS in PBS) and stained for 20 min at 4°C in the dark with anti-CD11c FITC (1∶50) and anti-B7.2 PE (1∶200) or anti-CD40 PE (1∶50) or anti-MHC II PE (1∶200) in the presence of Fc block (all reagents from BioLegend). Cells were washed with FACS buffer, fixed with 2% formaldehyde, and analyzed on a FACScan flow cytometer. The data was analyzed using WinMDI or Cell Quest Pro.

### Endocytosis analysis

Cy3-conjugated native P6 and non-lipidated rP6 were generated using the Cy3 Monoreactive Dye Pack (GE Healthcare). Following manufacturer's instructions, 1 mg of each antigen was filtered into 0.1 M Na_2_CO_3_/NaHCO_3_ buffer and mixed with Cy3 dye for 30 min at room temperature. Free dye was separated from the protein by filtration into PBS. Equivalent dye/protein ratios were determined by Lowry assay (Sigma). BMDCs were pulsed with 300 ng/ml of each conjugated antigen overnight. Harvested BMDCs were labeled with anti-CD11c FITC and acquired by two-color flow cytometry and ImageStream (Amnis) to determine the extent of endocytosis. For ImageStream analysis, cells were incubated with 100 µM LysoTracker Red (Invitrogen) for 30 min prior to harvesting and surface staining in order to visualize endocytosis and intracellular localization of Cy3-conjugated antigen.

BMDCs were washed once with medium, resuspended in complete medium at 2×10^5^ cell/ml and incubated either at 4°C or 37°C for 1 hr with FITC-Dextran 40,000 MW (Sigma) at a final concentration of 0.25 mg/ml. At the end of the incubation, cells washed three times with FACS buffer and stained for 20 min at 4°C in the dark with anti CD11c-PE (1∶400) in the presence of Fc block. Cells were washed with FACS buffer, fixed with 2% formaldehyde, and analyzed on a FACScan flow cytometer and WinMDI program. The values of the MFI at 4°C (background endocytosis) were subtracted from the MFI values obtained at 37°C.

### P6-specific T cell proliferation assay

WT or TLR2^−/−^ mice were immunized in the hind foot-pads with 25 µg of native P6 emulsified with CFA (Sigma) and boosted 1 week later with antigen in IFA (Sigma). One week after the second immunization the popliteal lymph nodes were collected and T cells were enriched over a mouse CD3^+^ enrichment column. Lymphocytes were seeded in round bottom 96 well plates with syngeneic irradiated BMDCs pulsed with the stimuli described above and co-cultured for 4 days. 1 µCi of ^3^H-thymidine (Perkin Elmer) was added to each well for the last 18 hrs of co-culture and the incorporation of ^3^H-thymidine was analyzed with a beta-counter.

### Cytokine ELISPOTs

Frequency of cytokine-secreting T cells from the spleen of P6-immunized mice was evaluated by cytokine ELISPOTs. Multiscreen Immobilon-P plates (Millipore) were coated overnight at 4°C with 3 µg/ml of rat anti-mouse IFN-γ (AN-18), anti-mouse IL-2 (JES6-1A12), or anti-mouse IL-4 (11B11). Lymphocytes were co-cultured with syngeneic irradiated BMDCs pulsed with the stimuli described above. After an 18 hr culture, the plates were washed extensively and cytokines were detected with biotinylated antibodies (IFN-γ, R4-6A2; IL-2, JES6-5H4; IL-4, BVD6-24G2) followed by addition of streptavidin-HRP. Spots were developed with tetramethylbenzidine (TMB) substrate and enumerated using a dissecting microscope.

### Induction and detection of anti-P6 antibodies

WT and TLR2^−/−^ mice were immunized intraperitonealy with 40 µg of native P6 or non-lipidated rP6 emulsified in CFA and boosted one week later with antigen in IFA and two weeks later with antigen alone. In some instances WT mice immunized with non-lipidated rP6 also received 40 µg of recombinant Pam3Cys (Invivogen) during each of the three immunization injections. Mice were bled retro-orbitally on a weekly basis. Titers of P6-specific antibodies and the associated Ig isotype and IgG subclasses were detected via an indirect ELISA, as described previously [9], [13].

### Statistical analysis

The values are representative of 3 or more independent experiments (as indicated in the legend of the figures) ± S.E.M. Testing for differences between means was determined using either 1-way ANOVA or 2-way ANOVA with post-test comparisons, and Student *t*-test (as indicated). Analysis was performed using GraphPad Prism v5.

## Results

### Immunization with lipid expressing P6 results in high titers of anti-P6 Ig

**Figure 1:**
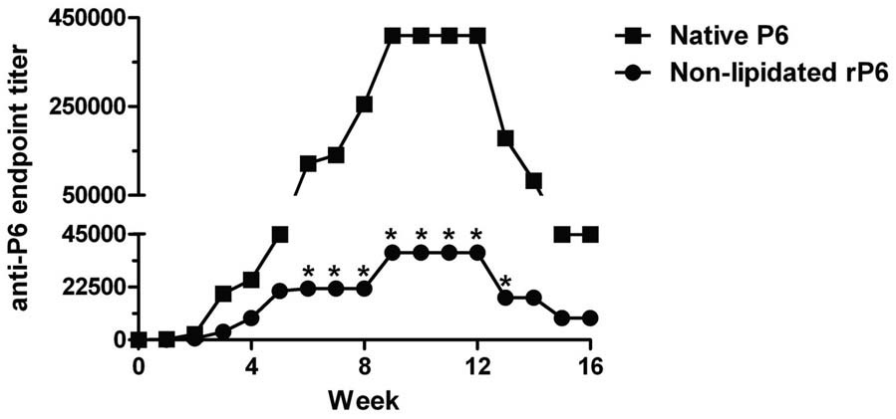
Anti-P6 Ig levels are elevated in mice immunized with P6 expressing lipid motif. Mice were immunized i.p. at one week intervals with 40 µg of native P6 (▪) or non-lipidated rP6 (•) emulsified in CFA, IFA, and PBS. Anti-P6 Ig levels were measured in pre-immune and post-immune sera by ELISA. Results are expressed as endpoint titers of serum dilutions. *p<0.05 2way ANOVA with Bonferroni post-test comparison between native P6 and non-lipidated rP6 at each timepoint.

The lipoprotein P6 is found in the bacterial outer membrane and is highly conserved among all NTHI strains, thereby making it an ideal protein target for vaccination studies. When extracted and purified directly from the bacterial outer membrane, it retains expression of the lipid motif. We first sought to determine whether the presence of this lipid motif impacts the generation of antibody titers following immunization. In order to validate the importance of lipid expression and its contribution to the immunogenicity of P6, we generated a recombinant version of P6 that lacks the N-terminal tri-palmitoyl lipid motif. The amino acid sequence of the recombinant P6 is 100% identical to native bacteria-purified P6. Naïve mice were immunized intraperitonealy with either native P6 or non-lipidated recombinant P6. Pre-immune and post-immune sera were collected from all mice and the magnitude and kinetics of antibody production was monitored (**Fig. 1**). Mice immunized with native P6 generated high titers of anti-P6 antibodies that were detectable as early as 2 weeks after initial immunization. The antibody titers reached peak levels 8 weeks after the first immunization. In contrast, the antibody titers in mice immunized with non-lipidated rP6 rose very modestly in the early weeks, even though the immunization schedule utilized adjuvant. Not only were the kinetics of anti-P6 Ig appearance slower in non-lipidated rP6 immunized mice, the magnitude of the antibody titers in the sera were substantially lower in comparison to mice immunized with native P6. The average peak antibody titers of mice immunized with non-lipidated rP6 was more than 10-fold lower than the level observed in mice immunized with native P6. This result suggests that the lipid motif and the nature of the immunizing protein play an important role in generating robust antibody titers.

**Figure 2:**
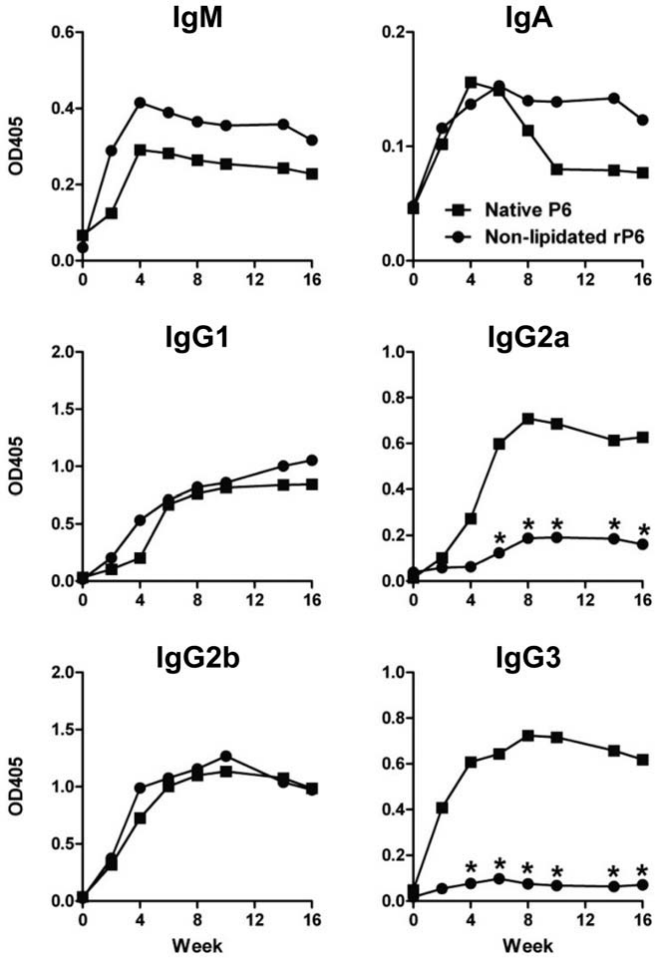
Anti-P6 Ig isotype and subclass analysis in immunized mice. Levels of Ig isotype and IgG subclass were measured by ELISA in WT mice immunized with native P6 (▪) or non-lipidated rP6 (•). OD values at 405 nm for sera dilutions at 10^−2.6^ (1∶400) were analyzed for levels of P6-specific IgA, IgM, IgG1, IgG2a, IgG2b, and IgG3. *p<0.05 2way ANOVA with Bonferroni post-test comparison between native P6 and non-lipidated rP6 at each timepoint.

In addition to measuring the kinetics and magnitude of anti-P6 Ig, we analyzed whether the difference in antibody response following immunization with non-lipidated rP6 could be accounted for by an alteration in the immunoglobulin subclass that is generated. The levels of IgM, IgA, and the four IgG subclasses that recognized plate-bound P6 were measured at a single serum dilution (**Fig. 2**). Mice immunized with native P6 did not display a bias in the repertoire of anti-P6 IgG subclasses generated, as all four IgG subclasses were present in mice at high levels. In contrast, mice immunized with non-lipidated rP6 displayed a striking bias in the IgG subclass of anti-P6 generated. Although antibodies of IgG1 and IgG2b subclasses were present at nearly equivalent levels in mice immunized with either non-lipidated rP6 or native P6, mice from the former group displayed low to negligible levels of IgG2a and IgG3 at all time points analyzed. Therefore, the presence of a lipid motif on an immunizing antigen can and does influence the subclass of antibody repertoire that is generated.

### Lipid motif on P6 augments T cell proliferation and cytokine production

**Figure 3:**
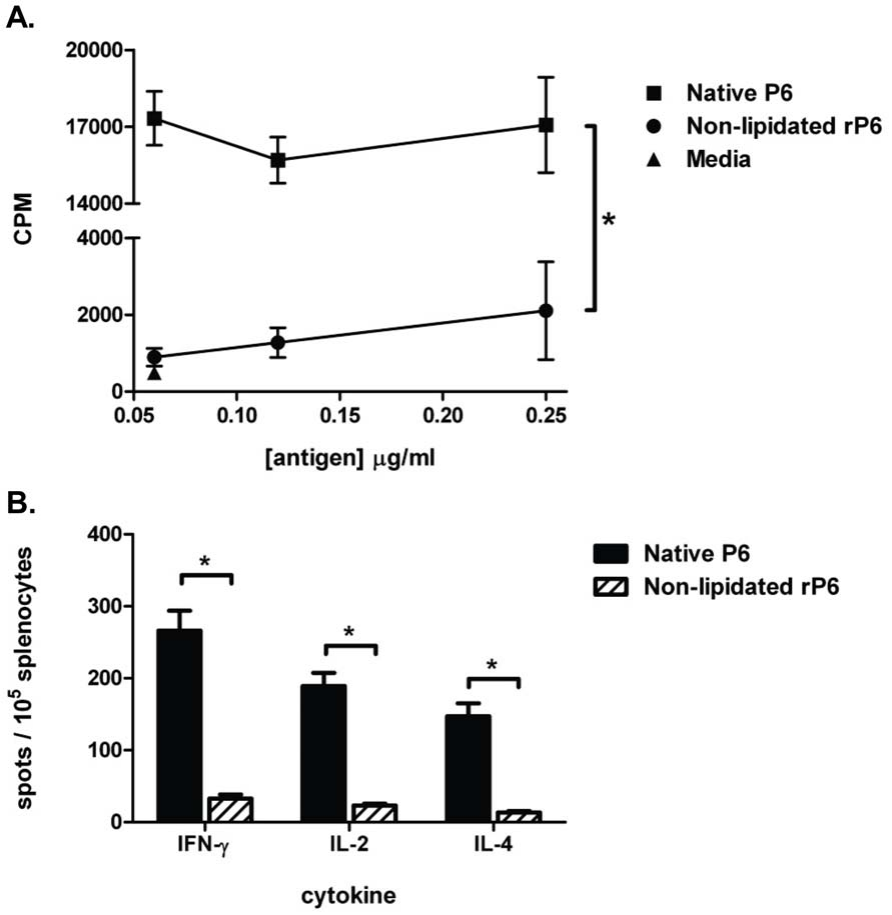
Lipid motif on P6 augments T cell proliferation and cytokine production. WT mice were immunized s.c. with 40 µg of native P6 emulsified in CFA and IFA one week later. (**A**) Proliferation of CD3^+^ cells isolated from draining lymph nodes was measured following 4 day co-culture with syngeneic irradiated BMDCs pulsed with 0.25, 0.12, and 0.06 µg/ml native P6 (▪) or non-lipidated rP6 (•). Media alone control (▴) was performed for background proliferation of T cells. Thymidine was added to the wells for the last 16 hrs of incubation. (**B**) Splenocytes from the same animals were co-cultured overnight with 0.06 µg/ml antigen pulsed irradiated BMDCs in ELISPOT plates coated with anti-cytokine mAb. Plates were developed and spots enumerated microscopically. *p<0.01 1way ANOVA with Bonferroni post-test comparison of native P6 to non-lipidated rP6.

The known requirement for T cell help in antibody class-switching prompted us to evaluate the effect of the lipid motif in antigen-specific T cell stimulation. Mice were immunized with native P6 and then *in vitro* T cell responses were measured against APC presenting either native P6 or non-lipidated rP6. CD3^+^ lymph node cells were isolated and co-cultured with syngeneic BMDCs pulsed with native P6 and non-lipidated rP6. Proliferation of T cells *in vitro* by co-culture with antigen-loaded APCs was measured by ^3^H-thymidine incorporation (**Fig. 3A**). BMDCs pulsed with non-lipidated rP6 provided minimal stimulation of antigen-specific T cells, whereas BMDCs pulsed with native P6 stimulated T cell proliferation at high levels.

The role of the lipid motif in eliciting cytokine production from the activated T cells was measured by cytokine ELISPOT (**Fig. 3B**). Splenocytes from native P6 immunized mice were harvested and cultured overnight with syngeneic irradiated BMDCs pulsed with either native P6 or non-lipidated rP6. In concordance with the data from the proliferation assay, BMDCs pulsed with non-lipidated rP6 were incapable of stimulating high numbers of antigen-specific T cells to secrete cytokines. These results lend further support to the finding that the lipidated antigen is a strong activator of T cells.

### Expression of TLR2 on APCs mediates responses to lipoprotein P6

**Figure 4:**
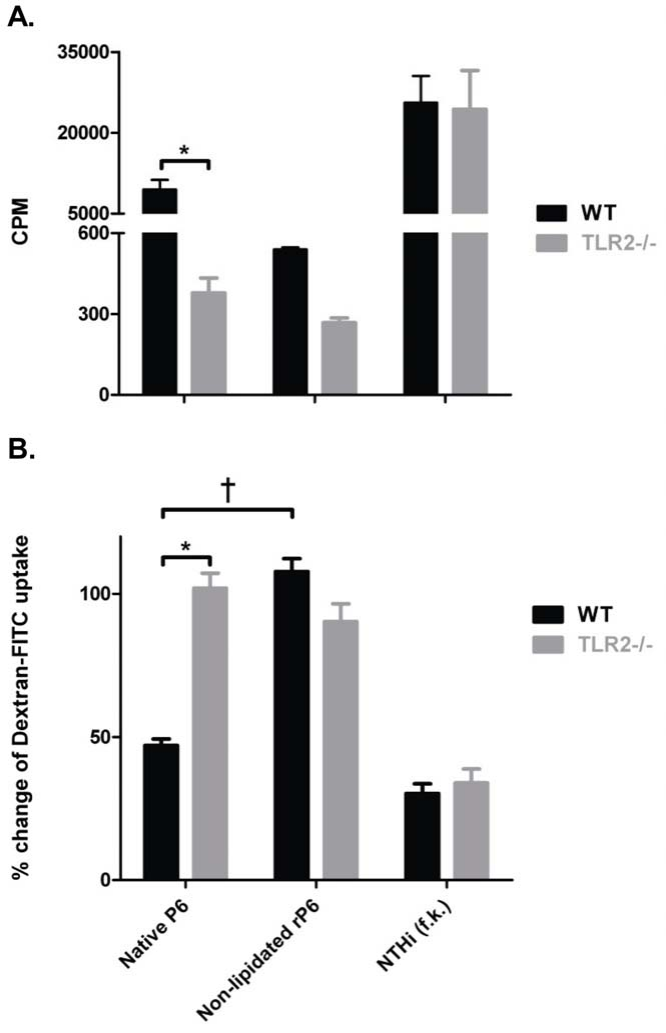
Expression of TLR2 on APCs mediates responses to lipoprotein P6. WT mice were immunized s.c. with 40 µg of native P6 emulsified in CFA and IFA one week later. (**A**) Proliferation of CD3^+^ cells isolated from draining lymph nodes was measured following 4 day co-culture with syngeneic irradiated WT (black) and TLR2^−/−^ (gray) BMDCs pulsed with 0.06 µg/ml native P6 or non-lipidated rP6. Thymidine was added to the wells for the last 16 hrs of incubation. (**B**) BMDCs from WT (black bar) and TLR2^−/−^ (gray bar) mice were incubated for 1 hr with the indicated stimuli and Dextran-FITC simultaneously (formalin-killed NTHI, f.k.). Cells were harvested and stained with anti-CD11c PE and acquired by two-color flow cytometry (FITC vs PE) to determine endocytic uptake of Dextran-FITC. Results are expressed as percent change in FITC MFI from media control. *p<0.01 1way ANOVA with Bonferroni post-test comparison of WT to TLR2^−/−^.

The striking differences between the ability of non-lipidated rP6 and native P6 to stimulate T cells and B cells prompted us to next examine what role the lipid motif might play on maturation and activation of dendritic cells. As TLR2 binds bacterial lipid motifs, we examined whether this receptor plays a role in the responses of DCs to P6. WT mice were immunized with native P6 and CD3^+^ T cells from lymph nodes were isolated and co-cultured with syngeneic irradiated WT or TLR2^−/−^ BMDCs pulsed with native P6, non-lipidated rP6, or formalin-killed NTHI (NTHI f.k.). Formalin-killed NTHI was utilized as a positive control in order to evaluate the capacity of TLR2^−/−^ APC to respond to ligands of NTHI that signal via other TLRs. WT BMDCs presenting native P6 were capable of eliciting T cell proliferation, whereas TLR2^−/−^ BMDCs stimulated with either native P6 or non-lipidated rP6 were unable to stimulate T cell proliferation (**Fig. 4A**). Importantly, this was not a global deficiency caused by the absence of TLR2, as both WT and TLR2^−/−^ BMDCs pulsed with formalin-killed NTHI induced T cell proliferation at similar levels. Expression of TLR2 on DCs therefore plays an essential role in the ability of these cells to respond to the lipid motif on P6 and activate antigen-specific T cells.

Uptake of antigen is an early step in antigen-processing and maturation of DCs. Immature DCs are highly endocytic and progressively lose this ability during their maturation process. The ability of DCs treated with native P6 to stimulate antigen-specific T cells prompted us to evaluate how recognition of P6 via TLR2 also affects the maturation of DCs. Endocytosis of FITC-labeled dextran was measured in stimulated BMDCs (**Fig. 4B**). APC that are matured by a potent stimulus will not endocytose FITC-dextran particles efficiently, whereas a stimulus that does not mature the BMDCs will allow the cells to continuing sampling the microenvironment. Native P6 stimulated WT BMDCs did not endocytose FITC-dextran to a high degree, revealing the potent maturation signal delivered by exposure to this stimuli. This mature phenotype of low endocytosis is attributable to the interaction of TLR2 with its ligand, as TLR2^−/−^ BMDCs exposed to native P6 continue to maintain high endocytic capacity. TLR2^−/−^ BMDCs could mature in the presence of other bacterial ligands expressed by formalin-killed NTHI and therefore exhibited reduced endocytosis of FITC-dextran. These experiments reveal that recognition of lipoprotein P6 by TLR2 is essential for DC maturation, as absence of either the ligand (i.e. lipid motif) or the receptor (i.e. TLR2) did not confer a potent maturation signal for APC.

### Lipid motif promotes enhanced uptake of P6 by APCs

**Figure 5:**
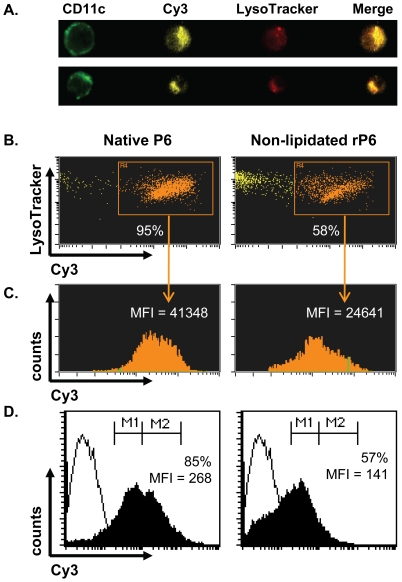
Enhanced endocytosis of lipid-expressing P6 by BMDCs. BMDCs from WT mice were incubated overnight with Cy3-conjugated native P6 or non-lipidated rP6. Cells were incubated with 100 µM LysoTracker Red for 30 min prior to harvest and then stained with anti-CD11c FITC. (**A**) Representative image analysis of Cy3-conjugated endocytosis and co-localization with LysoTracker for bacterial P6 (top row) and non-lipidated rP6 (bottom row). (**B**) Percent of CD11c^+^ BMDCs that endocytosed Cy3-conjugated antigen and co-localize with LysoTracker. (**C**) Amount of endocytosed antigen present in Cy3^+^ cells as determined by Cy3 mean fluorescent intensity (MFI). (**D**) Antigen uptake by BMDCs determined by two-color flow cytometry (FITC vs Cy3). Percent of CD11c^+^ cells that have endocytosed Cy3-conjugated antigen over unstimulated BMDCs are provided in each plot, in addition to MFI.

The difference in the ability of native P6 and non-lipidated rP6 to stimulate T cells is dependent on the maturation state of the APC and also the amount of antigen that has been taken up by the APC. We next evaluated whether the lipid motif might influence the extent of antigen uptake by DC and its intracellular localization. The level of antigen uptake was quantified using Cy3-conjugated of native P6 and non-lipidated rP6 by Amnis ImageStream and flow cytometry (**Fig. 5**). Endocytosis of native P6 was observed in 95% of BMDCs compared to 58% of BMDCs that had taken up non-lipidated rP6 (**Fig. 5B**); these values were additionally corroborated by flow cytometric analysis. Additionally, native P6 was endocytosed at nearly twice the amount of non-lipidated rP6 as measured by Cy3 fluorescent intensity (MFI) in both ImageStream (**Fig. 5C**) and flow cytometry analysis (**Fig. 5D**). The absence of the lipid motif did not alter the localization of the antigen in the endosomal compartment as determined by their equivalent co-localization pattern with lysotracker (**Fig. 5B**). Therefore, the stimulatory capacity of native P6 can be accounted for by its enhanced uptake in DCs.

### TLR2 expression mediates co-stimulatory molecule upregulation in response to P6

**Figure 6:**
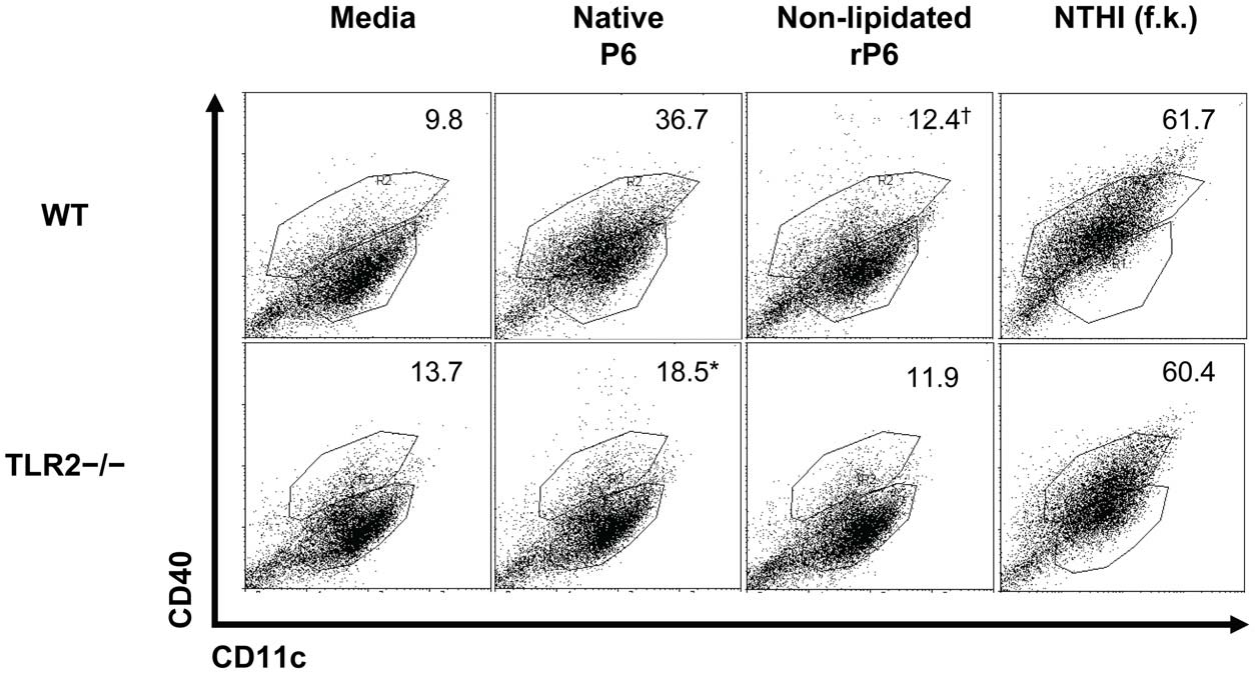
TLR2 expression on APC mediates upregulation of CD40 in response to lipoprotein P6. BMDCs from WT (top row) and TLR2^−/−^ (bottom row) mice were incubated overnight with the indicated stimuli. Cells were harvested and stained with anti-CD11c and anti-CD40 and acquired by two-color flow cytometry (FITC vs PE) to determine CD40 expression patterns. Percent of cells expressing CD40 over isotype are provided in each plot. *p<0.05 2way ANOVA with Bonferroni post-test comparison of native P6 between WT and TLR2^−/−^. ^†^p<0.05 2way ANOVA with Bonferroni post-test comparison of native P6 and non-lipidated rP6 in WT.

In order to activate naïve T cells, APC must upregulate surface expression of co-stimulatory molecules. We next examined what role the lipid motif of P6 and signaling via TLR2 played in the ability of P6-stimulated DC to express co-stimulatory molecules. BMDCs from WT and TLR2^−/−^ mice were cultured with native P6 and non-lipidated rP6 overnight. Relative surface expression levels of CD40 (**Fig. 6**) and CD86 was evaluated via two-color flow cytometry. An approximate two-fold increase in the percent of CD40^+^ cells was observed in native P6 stimulated WT BMDCs compared to TLR2^−/−^ BMDCs. Neither WT or TLR2^−/−^ BMDCs upregulated CD40 expression when stimulated with non-lipidated rP6, thus demonstrating the synergy between lipoprotein and TLR2 signaling for APC maturation. Data from relative surface expression of CD86 revealed a far greater dependence on the lipid motif and presence of TLR2 signaling (**Table 1**). The absence of the lipid motif on rP6 resulted in lack of CD86 upregulation, compared to native P6. TLR2^−/−^ BMDCs were incapable of upregulating CD86 in response to any form of the P6 protein, but were responsive to formalin-killed NTHI which expressed other TLR ligands.

**Table 1:** Co-stimulatory molecule expression on BMDCs.^a^

		Media	NativeP6	Non-lipidatedrP6	NTHI (f.k.)
**CD40**	**WT**	09.4±0.2	024.9±01.3*	11.5±0.8†	041.4±03.8
	**TLR2^−/−^**	09.3±0.1	012.0±01.7*	08.8±0.3†	035.0±08.8
**CD86**	**WT**	26.8±2.8	100.7±12.7*	39.9±5.6†	254.0±67.9
	**TLR2^−/−^**	30.9±2.3	038.1±03.7*	29.3±1.7†	200.9±39.3

a Mean fluorescence intensity ± SEM of anti-CD40 and anti-CD86 staining on WT and TLR2^−/−^ BMDCs.

*p<0.05 2way ANOVA with Bonferroni post-test comparison of native P6 between WT and TLR2^−/−^.

† p<0.05 2way ANOVA with Bonferroni post-test comparison of bacterial P6 and non-lipidated rP6 in WT.

Secretion of inflammatory cytokines IL-6 and TNF-α by stimulated BMDCs provided the most striking evidence supporting the requirement of both the lipid motif and TLR2 for APC maturation. Levels of cytokines from stimulated BMDCs were measured by Luminex multi-array (**Table 2**). WT and TLR2^−/−^ BMDCs produced low levels of IL-6 and TNF-α in the presence of media control. Native P6 induced potent secretion of inflammatory cytokines from WT BMDCs. Although stimulation with non-lipidated rP6 resulted in secretion of IL-6 and TNF-α above background, the amount of cytokine secreted was 10-fold lower compared to the levels from cells stimulated with native P6. Expression of TLR2 on the BMDC was important for secretion of IL-6 and TNF-α, as TLR2^−/−^ BMDCs did not secrete these cytokines to the same extent as WT BMDCs. Further, in the absence of both the lipid motif and TLR2, BMDCs were unable to secrete either of the cytokines assayed. However, TLR2^−/−^ BMDCs were able to produce cytokines when stimulated with formalin-killed NTHI, most likely due to the presence of LOS a known TLR4 ligand. P6-mediated inflammatory cytokine secretion is therefore dependent on the presence of a lipid motif on P6 and the ability of APC to recognize this motif via TLR2.

**Table 2:** Inflammatory cytokine secretion by BMDCs.^a^

		Media	NativeP6	Non-lipidatedrP6	NTHI (f.k.)
**IL-6**	**WT**	77	15,000	1,004	91,050
	**TLR2^−/−^**	22	01,270	0,031	31,400
**TNF-α**	**WT**	19	01,130	0,127	02,395
	**TLR2^−/−^**	14	00,151	0,029	02,840

a IL-6 and TNF-α secretion (pg/ml) by WT and TLR2^−/−^ BMDCs.

### TLR2 expression is important for antibody and recall cytokine responses against P6

**Figure 7:**
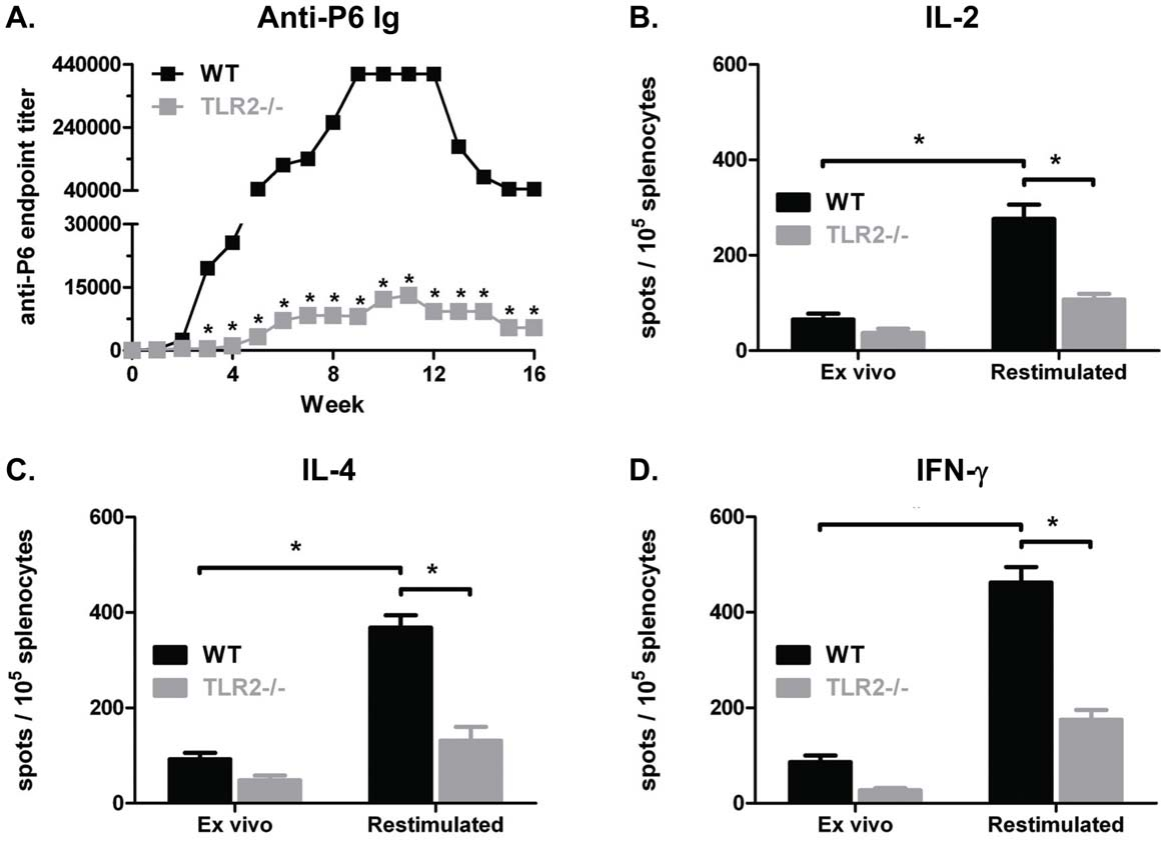
TLR2 expression is important for antibody and recall cytokine responses against P6. WT (black bar) and TLR2^−/−^ (gray bar) mice were immunized i.p. with 40 µg of native P6 emulsified in CFA, IFA, and PBS. (**A**) Anti-P6 Ig levels were measured in pre-immune and post-immune sera by ELISA. (**B–D**) Frequency of cytokine secreting T cells in spleens from the same animals after 16 weeks were measured by ELISPOT. Splenocytes were assayed directly *ex vivo* and after 3 day restimulation with BMDCs pulsed with native P6. Plates were developed and spots enumerated microscopically. *p<0.05 2way ANOVA with Bonferroni post-test comparison of WT and TLR2^−/−^.

The role of TLR2 in eliciting *in vivo* adaptive immune responses against P6 was evaluated. WT and TLR2^−/−^ mice were immunized with native P6, and the magnitude and kinetics of anti-P6 Ig in sera was monitored (**Fig. 7A**). WT mice produced very high titers of anti-P6 Ig following immunization, whereas anti-P6 antibodies made by TLR2^−/−^ mice were significantly lower at all time points. Therefore, it is reasonable to conclude that expression of the endogenous lipid motif on P6 and recognition of this motif by TLR2 is required for effective immunization. Splenocytes were assayed directly *ex vivo* 16 weeks after the initial immunization and also following a 3 day restimulation *in vitro* in order to measure the frequency of IFN-γ, IL-2, or IL-4-secreting P6-specific T cells by ELISPOT (**Fig. 7B–D**). The frequency of cytokine-secreting T cells directly *ex vivo* in both WT and TLR2^−/−^ mice was low, but expected, given the protracted time interval (16 weeks) between the last exposure to antigen *in vivo* and analysis *ex vivo*. Splenocytes from WT mice exhibited robust recall responses when restimulated *in vitro* with P6, as the frequency of IFN-γ, IL-2, and IL-4 secreting T cells increased. In contrast, splenocytes from TLR2^−/−^ mice exhibited poor recall responses even following *in vitro* restimulation with P6. These data reinforce the notion that optimal adaptive immune responses to the P6 antigen require the presence of the lipid motif on the lipoprotein and the cognate receptor (i.e. TLR2) for transduction of the signal.

### Direct conjugation of lipid motif on P6 is important for induction of high titers of anti-P6 antibodies

**Figure 8:**
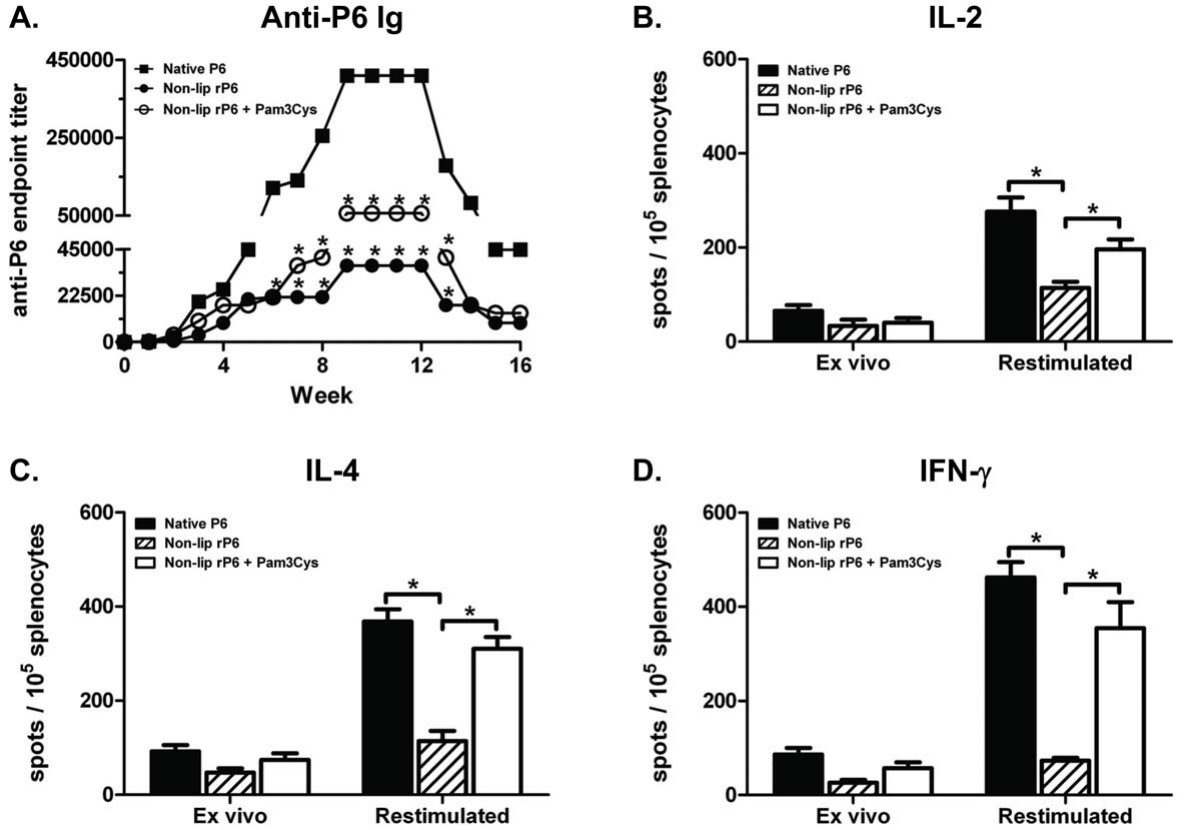
Direct conjugation of lipid motif on P6 is important for induction of high titers of anti-P6 antibodies and cytokine secretion by antigen-secretion T cells. WT mice were immunized i.p. with 40 µg of native P6 (▪), non-lipidated rP6 (•), or non-lipidated rP6 plus Pam3Cys (open circle) emulsified in CFA, IFA, and PBS. (**A**) Anti-P6 Ig levels were measured in pre-immune and post-immune sera by ELISA. (**B–D**) Frequency of cytokine secreting T cells in spleens from the same animals after 16 weeks were measured by ELISPOT. Splenocytes were assayed directly *ex vivo* and after 3 day restimulation with BMDCs pulsed with native P6. Plates were developed and spots enumerated microscopically. *p<0.05 2way ANOVA with Bonferroni post-test comparison.

Based on the complementary results obtained from immunization with non-lipidated rP6 in WT mice and native P6 immunization in TLR2^−/−^ mice, we reasoned that optimal stimulation with P6 required the direct conjugation of the lipid motif to the antigen. In order to test the necessity of having a directly conjugated lipid motif on P6 during immunization, non-lipidated rP6 was given to WT mice in the absence or presence of exogenous lipid, Pam3Cys. Equivalent amounts of Pam3Cys and non-lipidated rP6 were injected; separate emulsions of Pam3Cys and non-lipidated rP6 were generated but were injected simultaneously. With this experimental design, it is possible to directly test whether anti-P6 responses can be generated when the lipid motif is not conjugated to the vaccine antigen but is present in the vaccine formulation. Data from **Figure 1** is presented again in **Figure 8A** for ease of comparing to the anti-P6 Ig titers obtained in mice immunized with non-lipidated rP6 plus Pam3Cys to mice immunized with non-lipidated rP6 alone. The presence of Pam3Cys at the time of immunization with non-lipidated rP6 elicited higher titers of total anti-P6 compared to immunization without the lipid motif. The levels did not approach those observed in native P6 immunization, but suggested that the presence of the exogenously added lipid motif partially rescued the diminished titers resulting from immunization with non-lipidated rP6 alone. Additionally, the repertoire of anti-P6 IgG subclasses was similar in mice immunized with native P6 or non-lipidated rP6 plus Pam3Cys (data not shown). Therefore, the presence of the Pam3Cys motif during immunization with the non-lipidated antigen is able to enhance the magnitude and the nature of the antibody response. Along with the enhancement of antibody responses, the presence of the recombinant lipid motif generated unbiased cytokine-secreting T cells at similar frequencies as in mice immunized with native P6 (**Fig. 8B–D**). Collectively our data establish that the presence of the lipid motif is crucial for robust antibody responses to P6 and these responses are optimally generated when the motif is directly conjugated to the protein antigen.

## Discussion

In this study, we undertook a systematic analysis of the contribution of the lipid motif of lipoprotein antigen P6 to its ability to stimulate both innate and adaptive immune responses. As this lipoprotein is a key component required for maintaining the cellular integrity of the outer membrane in NTHI, it is to no surprise that P6 is highly conserved among several NTHI strains [8]. Furthermore, several lines of evidence indicate that P6 induces protective immune responses in humans indicating that the protein is a promising vaccine candidate for otitis media and COPD. In evaluating the role of the lipid motif in P6, our studies have highlighted the pivotal role of this motif in determining the immunogenicity of P6.

It is clear that TLR ligands help shape immune outcomes, both in the setting of vaccination and infection. In addition to creating robust immune responses, TLR ligands are also involved in the skewing of protective immunity upon vaccination. Using a recombinant Salmonella vaccine in MyD88^−/−^ mice, it was shown that CD4^+^ T cells and iNKT cells were impaired in their ability to provide help for Th1-dependent antibody responses [15]. The effect of TLR ligation is often observed in the programming of DC towards a phenotype that stimulates naïve T cells and secretion of pro-inflammatory cytokines. The P6 vaccine antigen we have investigated demonstrates a unique ability to induce non-skewed adaptive immune responses via the activation of stimulatory DC.

Immunization with purified native P6 elicited very robust titers of antibodies that were durably sustained (up to 16 weeks) after the initial antigen injection and the high antibody titers reveal the potent immunogenicity of this lipoprotein. The repertoire of IgG subclasses elicited by native P6 suggests that not only was the immunization robust but also the response was unbiased, as all four major IgG subclasses were present at substantial levels in the sera. Unbiased antibody repertoire generation is an appealing feature of a bacterial vaccine antigen. Activation of TLR signals on DC during vaccination instruct the generation of effector and memory lymphocytes [1], [16]. The phenotype and TLR expression pattern of the DC that responds to the NTHI infection may skew the response to either Th1 or Th2, thus a vaccine that can elicit both types of memory T cells will be ideally suited for a rapid response in the face of infection. Ligands for TLR2 have been shown to stimulate both Type 1 (e.g. IgG2a) and Type 2 (e.g. IgG1) immune responses [17]–[21]. Generation of skewed antibody responses elicited by non-lipidated rP6 probably reflect the contribution of the protein component. The use of Pam3Cys as an adjuvant is often associated with Type 2 responses, although other groups have also demonstrated the production of IFN-γ Type 1 responses [22], [23].

The immunogenicity of P6 is facilitated by the presence of a CD4 helper T cell epitope [13], [24], [25]. The presentation of the epitope to P6-specific CD4^+^ T cells is likely enhanced by the presence of the lipid motif on the antigen when endocytosed by the APC upon recognition by TLR2. Using a fluorophore-conjugated P6, enhanced rates of endocytosis were observed for the lipid motif-expressing antigen; thus TLR2-mediated targeting of the antigen impacts on the amount of antigen that an APC is likely to process and present to antigen-specific T cells. This ensures that the DC which endocytose the P6 antigen mature into a professional APC capable of presenting the helper epitope. Thus, antigen presentation of the helper epitope is localized in the same APC that has been stimulated by the TLR2 ligand. There was the theoretical possibility that other potential TLR2 ligands, such as peptidoglycan [26], which could be present in very small contaminating quantities during P6 purification could potentially contribute to the robust production of IL-6 in TLR2 expressing DCs. However, our experimental approach using both the TLR2-deficient APCs and non-lipidated rP6, would strongly suggest that the immunostimulatory capacity of bacterial P6 is dominated by this antigen and not other contaminating molecules during purification.

Lipopeptides have been utilized as adjuvants in several vaccine models [27]–[31]. This is due to their ability to bind TLRs present on antigen presenting cells, leading to their maturation and subsequent activation of helper T cells and antibody producing B cells. Adjuvant use of lipopeptides elicits both Th1 and Th2 cytokines depending on the model antigen used in immunizations [30], [32]. TLR-mediated activation of innate immune cells leads to the robust stimulation of adaptive immune lymphocytes, although the mechanism by which this occurs varies greatly in each individual model. The lipid motif on P6 is required for recognition by TLR2 expressed on DCs and leads to a robust stimulation of T cells *in vivo*. Activation of NF-κB signaling results from ligation of TLR2 by P6 and therefore accounts for the upregulation of surface co-stimulatory molecules and secretion of inflammatory cytokines [33], [34]. We have observed robust production of IL-6 and TNF-α from TLR2-expressing APCs stimulated with native P6, suggesting that NF-κB signaling is activated and leads to cytokine secretion. Production of these cytokines, among others, is critical for the activation of T cells.

TLR agonists are used as adjuvants in many vaccine formulations to enhance immune responses; however, the contribution of TLR signaling for the generation and maintenance of antibodies remains controversial. Pasare *et al* showed evidence for TLR requirement in eliciting an antibody response, but in a separate study, Gavin *et al* utilized mice devoid of TLR signaling to produce antibody responses against TLR ligand-free antigens [35], [36]. Recently, intrinsic TLR signals in B cells were demonstrated to enhance antibody responses, but were dispensable for their induction [37]. TLR signals could be involved at various points of B cell activation, through either directly signaling on the B cells during antigen recognition or indirectly following DC-mediated T helper responses. Though B cells require help from CD4^+^ T cells in order to initiate effector functions, such as immunoglobulin class switching, the expression of surface TLR2 on B cells could result in the direct activation of P6-specific B cells. The pattern of TLR expression varies on B cells and the nature of the ensuing response can differ from the response elicited by TLR-activated DCs [38], [39]. The immune modulation of TLR activation in B cells includes the secretion of cytokines that are not produced by DCs, such as IL-10 and IFN-γ [40], [41]. The direct activation of B cells during P6 immunization cannot be discounted and may explain the production of the four P6-specific IgG subclasses. The inclusion of TLR agonists has been shown to be crucial for the generation of protective anti-RSV antibodies following immunization with an inactivated vaccine [42]. TLR signaling by the B cells directly leads to the production of long-lived antibodies, suggesting that directing responses to B cells with TLR agonists may be important for efficacious immunizations. The lipid motif on P6 may stimulate T cells not only via DCs but also likely through B cell activation. Immunization with the MALP-2 lipopeptide has been shown to activate B cells directly via TLR2 in the absence of CD4^+^ T cell help. MALP-2 activated B cells were capable of secreting IgM and IgG, but required CD4^+^ T cell in order to secrete IgA [43]. Immunization with P6 likely activates the B cells directly due to the expression of TLR2 and this response is further enhanced due to the presence of DC stimulated CD4^+^ T cells.

The importance of direct conjugation of Pam3Cys on the P6 antigen during immunization was highlighted by the modest enhancement of antibody response when the TLR agonist was admixed with non-lipidated rP6. Thus, in spite of utilizing excess free Pam3Cys, the magnitude of antibodies generated to non-lipidated rP6 plus exogenous lipid motif was markedly lower than the response elicited to native P6 in which the lipid motif is directly conjugated to the protein. Other TLR agonists have been used as fusions or admixed with protein antigens for effective immunizations. Flagellin, the only known TLR5 ligand, has been conjugated to Listeria p60, influenza virus matrix and hemagglutinin in order to enhance immune responses to these antigens during vaccination [44]–[46]. Admixing of flagellin with *Plasmodium vivax* MSP-1 protein generated strong responses to this novel malaria vaccine [47]. Enhancement of immune responses with inclusion of flagellin in the vaccine formulation, either by direct conjugation or admixing, is likely due to its inherent stimulatory capacity. Pam3Cys has also been used as an adjuvant in admixing vaccine formulations; however, optimal responses to P6 require direct conjugation of this TLR ligand. Based on our results, an effective vaccine for NTHI that utilizes the conserved P6 protein in its formulation, should include the Pam3Cys lipid motif, preferably directly conjugated to the antigen, in order to elicit robust and sustained activation of innate and adaptive immunity.

In this study, we have identified the role and mechanism of action of the amino-terminal lipid motif on the lipoprotein P6 of NTHI and demonstrated that the presence of this moiety on the antigen during immunization resulted in production of high titers of serum anti-P6 and robust activation of T cells. Lipidated P6 matured TLR2 expressing DCs, rendering these cells into potent APC for P6-specific T cells. Recognition of the lipid motif *in vivo* by TLR2 was also critical for the effective generation of anti-P6 antibodies and T cells. Taken together, this work has detailed the underlying basis for the enhanced immunogenicity of the P6 lipoprotein. This notable feature of P6 can therefore be exploited to improve vaccine formulations that utilize P6 for protection against NTHI infections.
